# A prospective study to correlate the cervical length by transvaginal ultrasound with preterm labor and perinatal outcomes

**DOI:** 10.1016/j.xagr.2025.100561

**Published:** 2025-08-14

**Authors:** Nina Mahale, Devangi Panchal, Ajit Mahale, Sonali Ullal, Merwyn Fernandes, Sonali Prabhu

**Affiliations:** 1 Departments of Obstetrics and Gynaecology (N Mahale and Panchal); 2 Radiodiagnosis (A Mahale, Ullal, Fernandes, and Prabhu), Kasturba Medical College Mangalore, Manipal Academy of Higher Education, Manipal, India.

**Keywords:** cervical, labor, outcome, perinatal, preterm, transvaginal, ultrasound

## Abstract

**BACKGROUND:**

This study aimed to investigate the role of transvaginal ultrasound as a screening tool to assess cervical length and improve perinatal outcomes to prevent preterm labor through appropriate interventions.

**OBJECTIVE:**

This study aimed to determine the mean cervical length at 11 to 14 and 18 to 22 weeks of gestation and to estimate the rate of cervical length shortening and its correlation with birth outcomes.

**STUDY DESIGN:**

This prospective study was conducted at Lady Goschen Hospital and KMC Hospital, Attavar, both of which are affiliated with Manipal Academy of Higher Education. This study included 93 pregnant women who came for antenatal care at Lady Goschen Hospital and KMC Hospital, Attavar, from December 2022 to August 2024. All participants provided consent to participate in the study. The formula is a follows: n = Zα2 (Sn)(1-Sn) / L2 × p, where Zα = 1.96 at a 95% confidence level, Sn = sensitivity, l = allowable error, and p = negative prevalence (Sn = 75% [reference article], L = 20% [80% power], and p = 50% (assumption) with 95% confidence interval and 80% power with respect to reference [prediction of preterm labor by cervical length]). Convenient sampling was performed. Data analysis was performed using descriptive statistics. Changes in follow-up were estimated using analysis of variance and the Bonferroni *t* test. Sensitivity, specificity, positive predictive value, and negative predictive value were estimated. Statistical analysis was performed using SPSS (version. 2.0; IBM, Armonk, New York). A *P* value of >.05 was considered statistically significant.

**RESULTS:**

Cervical length measured using transvaginal ultrasound at 18 to 22 weeks of gestation was found to be more sensitive and to have better positive predictive value in predicting preterm labor than cervical length measured at 11 to 13 weeks of gestation. Cervical length between 18 and 22 weeks of gestation and the reduction of cervical length were found to be statistically significant in predicting preterm labor (*P*=.001).

**CONCLUSION:**

Transvaginal ultrasound is a noninvasive and comparatively affordable modality for identifying women at risk of preterm labor. In addition, this technology could help identify women at risk of preterm labor, thereby reducing morbidity and mortality.

**AJOG Global Reports at a Glance**

**Why was this study conducted?**

This study aimed to assess transvaginal ultrasound (TVS) as a diagnostic modality for cervical length CL discrepancy and its role in perinatal outcomes.

**Key findings**

TVS was used to determine the CL measurement at 18 to 22 weeks of gestation and the reduction of CL. Moreover, TVS was determined to be a sensitive and inexpensive test for predicting preterm labor.

**What does this add to what is known?**

Routine use of TVS as a CL screening test improves perinatal outcomes by preventing preterm labor through appropriate interventions.

## Introduction

Preterm birth (PTB) is defined as “birth of live neonates before 37 weeks of completed gestation.” Based on gestational age, it is categorized into “extremely PTB (<28 weeks of gestation), early PTB (28–32 weeks of gestation), and late PTB (32–37 weeks of gestation).” In India, approximately 9% of neonates born every year are preterm.^1^^,^^2^ Cervical length (CL) assessment may detect preliminary changes and may be beneficial for preventive measures of PTB.^3^ Early PTB is one of the main reasons for neonatal deaths, neonatal intensive care unit (NICU) admissions, and neurologic disabilities.^4^ Approximately 75% of neonatal deaths are accounted for by PTB.^5^ Risk factors include a previous history of preterm delivery, cervical incompetence, premature rupture of membranes (PROM), chorioamnionitis, preeclampsia, and eclampsia. Short CL is one of the major risk factors for preterm delivery.^6^ It is defined as “CL below the 10th percentile for gestational age. It corresponds to <25 mm at 18 to 24 weeks of gestation.” It predisposes the patient to cervical incompetence, chorioamnionitis, and PROM, which in turn leads to preterm labor. Hence, determining CL in early pregnancy is important to treat at-risk mothers. It can be determined by per vaginum examination, transvaginal, transabdominal, transperineal ultrasonography, or magnetic resonance imaging. Transvaginal ultrasound (TVS) is an easy and inexpensive method to accurately assess the CL at a routine antenatal check-up visit, which could help to determine the risk of preterm delivery in both singleton and multiple pregnancies.^7^ Some studies measured CL at 11 to 14 weeks of gestation,^8^^,^^9^ but other studies determined CL as a reliable predictor at 20 to 22 weeks of gestation.^8^ Keeping this background in mind, we have studied the correlation between PTB and CL measured using TVS, mean CL at different gestational time points, and associated birth outcomes.

### Aims

This study aimed to study the correlation between PTB and CL measured using TVS and to study the birth outcomes.

### Objectives

This study aimed to determine the mean CL at 11 to 14 and 18 to 22 weeks of gestation and to estimate the CL shortening rate and its correlation with birth outcomes.

## Materials and methods

This study included 93 pregnant women who came for antenatal care at or before 11 weeks of gestation at Lady Goschen Hospital and KMC Hospital, Attavar, Mangalore, and gave their consent to be included in the prospective study conducted by us from December 2020 to August 2022. Patients with multiple pregnancies, fetal anomalies, and uterine anomalies were excluded. CL was measured at 11 to 13 weeks of gestation during the routine nuchal translucency scan. The patients were followed up, and repeat CL was measured at 18 to 22 weeks of gestation by TVS during a routine anomaly scan.

**Figure 1:**
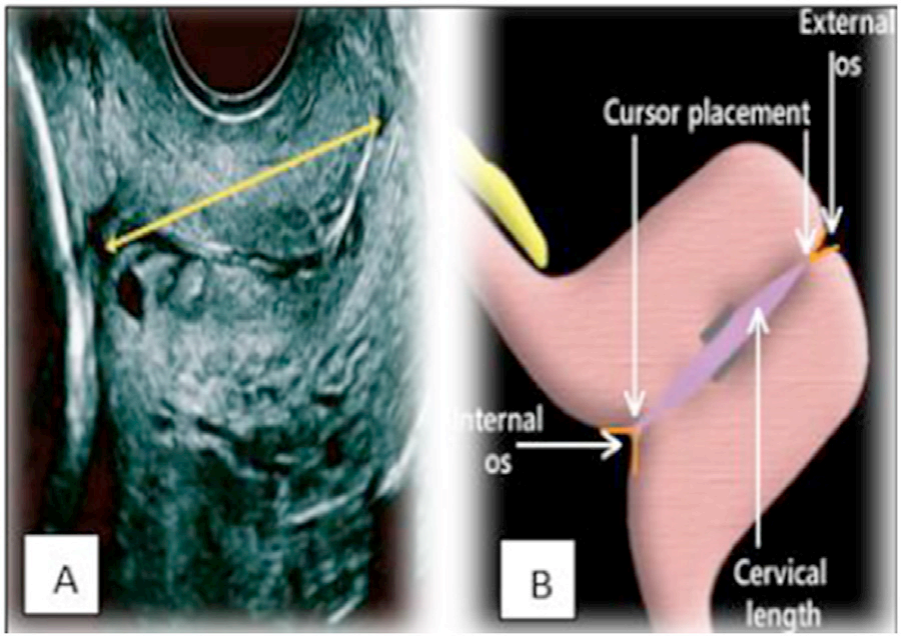
A, Transvaginal ultrasound image. B, Diagrammatic image of measurement of cervical length from the external os to the internal os

**Figure 2:**
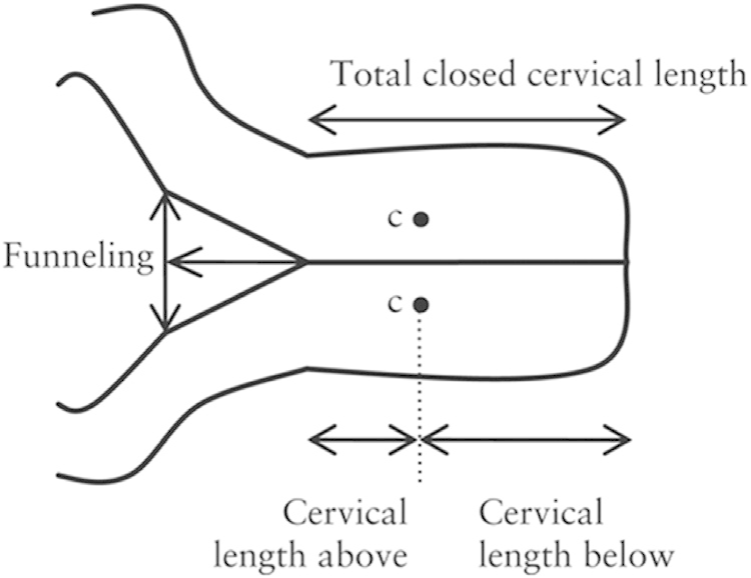
Schematic representation of transvaginal ultrasnographic cervical measurements

**Figure 3:**
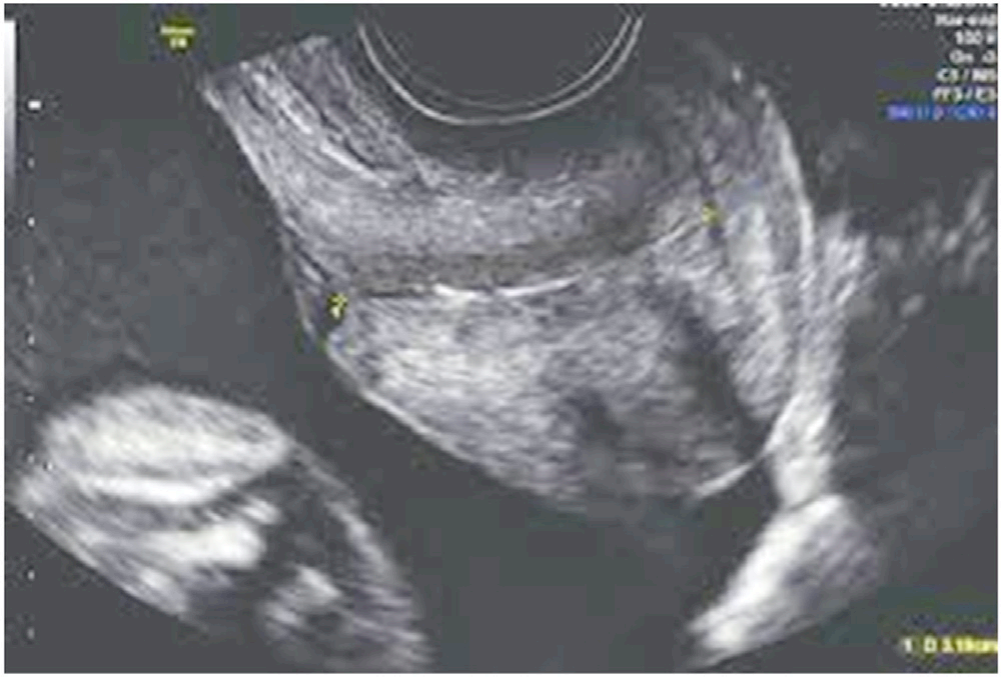
Determination of cervical length by ultrasound

The CL is conventionally measured using TVS. Here, after obtaining the informed consents, the women were asked to fully empty their urinary bladder. The woman is usually placed in a dorsal position and a transvaginal probe covered with a lubricated condom is inserted and guided by the anterior vaginal fornix. The sagittal view of the endocervical canal in the long axis along with the CL can be visualized. A high-frequency endovaginal probe (5–7 MHz) is generally used for this process. The length of the cervix from the external os to the internal os is measured along with the endocervical canal. In addition, certain anatomic cervical changes, such as funneling, funnel length, width index, and anterior and posterior cervical width, are recorded simultaneously (Figure 1, Figure 2, Figure 3).

Mahale. Transvaginal ultrasound in cervical evaluation for perinatal outcomes. Am J Obstet Gynecol Glob Rep 2025.

Mahale. Transvaginal ultrasound in cervical evaluation for perinatal outcomes. Am J Obstet Gynecol Glob Rep 2025.

Mahale. Transvaginal ultrasound in cervical evaluation for perinatal outcomes. Am J Obstet Gynecol Glob Rep 2025.

The difference between the 2 depicted the reduction of CL, and the patients were categorized into 3 groups based on the reduction of CL measured during 11to 13 and 18 to 22 weeks of gestation: group A, reduction in CL of ≤0.5 cm; group B, reduction in CL of 0.5 to 1.0 cm; and group C, reduction in CL of ≥1.0 cm. The patients were followed up until delivery to determine the birth outcomes.

## Results

Most patients (37.63%) belonged to the age group of 21 to 25 years. The youngest patient was 18 years, and the oldest patient was 42 years. Most patients (50.54%) enrolled were multigravida. Of note, 2 patients enrolled had recurrent abortion history (Table 1). Most patients had CL between 4.1 and 4.5 cm. Here, the minimal and maximum CLs recorded were 2.1 and 4.9 cm, respectively. The cutoff value for PTBs was 3.7 cm, above which PTBs were not observed. The mean CL was 4 cm (Table 1).

**Table 1:** Patient distribution according to the cervical length between 11 and 13 weeks of gestation on transvaginal ultrasound

Cervical length (cm)	No. of pregnant women	Percentage
2.6–3.0	3	3.22
3.1–3.5	16	17.20
3.6–4.0	17	18.28
4.1–4.5	46	49.46
4.6–5.0	11	11.83
Total	93	100.00

**Bar chart**

Most patients had a cervical length between 4.1 and 4.5 cm. The minimal cervical length recorded in the study was 3.0 cm, and the maximum cervical length noted was 5.0 cm. The cutoff value for preterm births was 4.2 cm, above which preterm births were not noted.

Most patients (38.7%) had a CL between 3.6 and 4.0 cm. The minimal CL recorded was 2.1 cm, and the maximum CL recorded was 4.9 cm. The patient with a CL of 2.1 cm had a spontaneous abortion. A patient with a CL of 2.2 cm was planned for cervical cerclage but underwent spontaneous abortion. The cutoff value for PTBs was 3.7 cm, above which PTBs were not observed. The mean CL was 3.45 cm (Table 2).

**Table 2:** Patient distribution according to the cervical length at 18 to 22 weeks of gestation by transvaginal ultrasound

Cervical length (cm)	No. of pregnant women	Percentage
2.1–2.5	3	3.22
2.6–3.0	23	24.73
3.1–3.5	17	1.83
3.6–4.0	36	38.70
4.1–4.5	11	11.83
4.6–5.0	2	2.15
Total	93	100.00

Most patients (55.91%) had a reduction in CL between 0.5 and 1.0 cm. The minimum and maximum reductions in CL were 0.2 and 1.2 cm, respectively. The minimum difference below which no PTB was observed was 0.4 cm (Table 3).

**Table 3:** Patient distribution according to reduction in cervical length

Reduction in cervical length	No. of patients	Percentage
Group A (<0.5 cm)	38	40.86
Group B (0.5–1.0 cm)	52	55.91
Group C (>1.0 cm)	3	3.23
Total	93	100.00

Here, 2 patients had spontaneous second-trimester abortions and had a reduction in CL of >1.0 cm. Among patients who had extreme preterm delivery, 1 had a reduction in CL of >1.0 cm, and 2 had a reduction in CL of 0.5 to 1.0 cm. Among 9 patients who had late preterm deliveries, 7 had a reduction in CL between 0.5 and 1.0 cm, and 2 had a reduction in CL of <0.5 cm (Table 4).

**Table 4:** Patient distribution as per gestation age

Gestational age at delivery	Group A (n=38) (reduction of <0.5 cm)	Group B (n=52) (reduction of 0.5–1.0 cm)	Group C (n=3) (reduction of >1.0 cm)
<28 wk	0	0	2
Early preterm (<34 wk)	0	2	1
Late preterm (34–37 wk)	2	7	0
Term gestation (≥37 wk)	36	43	0

Among patients with <2.5 kg, 3 had a reduction in CL of >1.0 cm, 13 had a reduction in CL of 0.5 to 1.0 cm, and 5 patients had a reduction in CL of <0.5 cm.

Here, 2 patients had spontaneous second-trimester abortions, where 1 patient was planned for a cervical encerclage. Moreover, 3 patients had extreme PTB, and 9 had late PTB. Overall, 79 patients had term deliveries (Table 5).

**Table 5:** Patient distribution as per the period of gestation at delivery

Period of gestation	No. of patients	Percentage
<28 wk	2	2
Early preterm (<34 wk)	3	3
Late preterm (>34 wk)	9	10
Term gestation (>37 wk)	79	85

Among 93 patients, 12 neonates needed NICU care. Moreover, 4 neonates needed NICU care for more than 7 days.

CL measured using TVS at 18 to 22 weeks of gestation was found to be more sensitive and to have better positive predictive value than CL measured at 11 to 13 weeks of gestation in predicting preterm labor.

CL between 18 and 22 weeks and the reduction of CL were found to be statistically significant in predicting preterm labor (*P*<.001) (Table 6).

**Table 6:** Diagnostic Indices

Test	Sensitivity	Specificity	PPV	NPV	*P* value
Cervical length at 11–14 wk	92.3%	55.0%	25.0%	97.2%	.002
Cervical length at 18–22 wk	100.0%	46.3%	23.2%	100.0%	<.001
Reduction in cervical length	69.6%	82.0%	52.3%	98.6%	<.001

## Discussion

### Principal findings

The CL measured at 11 to 14 weeks of gestation was not as sensitive in predicting PTBs, similar to the findings of Wadhawan et al^10^ and Kore et al.^11^

The duration of pregnancy directly correlated with the length of the cervix. Of note, a short cervix more often resulted in preterm delivery.^12^ Most patients (49.46%) in our study had a CL between 4.1 and 4.5 cm, which was similar to the study conducted by Carvalho et al.^13^ The cutoff value for PTBs was 3.7 cm, above which PTBs were not observed.

### Results

Here, most patients (37.63%) belonged to the age group of 21 to 25 years. The minimum age was 18 years, and the maximum age was 42 years. The median age of the patients was 25 years. Most patients (50.54%) were multiparous. The minimal and maximum CLs recorded were 2.1 and 4.9 cm, respectively.

### Clinical implications

Most patients (38.7%) had a CL between 3.6 and 4.0 cm. Here, the minimal and maximum CLs recorded were 2.1 and 4.9 cm, respectively. The mean CL was 3.5 cm. The patient with a CL of 2.1 cm had a spontaneous abortion. A patient with a CL of 2.2 cm was planned for cervical cerclage but underwent spontaneous abortion. The cutoff value for PTBs was 3.7 cm, above which PTBs were not observed. The CL measurement at 18 to 22 weeks of gestation was highly sensitive in predicting PTBs and was statistically significant.

Here, the maximum number of participants had a reduction in CL of 0.5 to 1.0 cm (ie, 55.91%).

### Research implications

Here, 85% of the participants had term deliveries, and 2% of the participants had spontaneous abortions. Moreover, 3% of the participants had early preterm delivery, and 10% of the participants had late preterm delivery. The minimum gestational age recorded was 24 weeks. Most patients in group C with a reduction in CL of >1.0 cm underwent preterm delivery. Of note, 2 patients had spontaneous second-trimester abortions. Moreover, 3 patients had early PTBs, with a CL between 3.1 and 3.5 cm at 11 to 14 weeks of gestation, a reduction in CL between 0.8 and 1.2 cm, and a CL between 2.1 and 3.5 cm at 18 to 22 weeks of gestation. Of note, 9 patients had late PTBs, with a CL of 3.1 to 3.5 cm at 11 to 14 weeks, a reduction ranging from 0.3 to 0.8 cm, and a CL of 2.8 to 3.5 cm at 18 to 22 weeks of gestation. The neonates of 100% of patients required an NICU stay. Among 93 patients, 2 had spontaneous abortions. Of note, most patients had vaginal delivery (60%). Of 93 patients, 35 underwent lower segment cesarean delivery (LSCD) given the following indications: previous LSCD not a candidate for trial of labor after cesarean (n=16), meconium-stained amniotic fluid (n=5), nonreassuring fetal heart status (n=4), cephalopelvic disproportion (n=2), malpresentation (n=3), failed induction (n=3), and cervical dystocia (n=2). Of 93 patients, 12 presented with PROM, and 7 presented with preterm PROM. Further studies may help improve perinatal outcomes.

### Strengths and limitations

Our study has several strengths. First, the sample size was adequately calculated, according to the mathematical calculations used in the methods. Second, we adhered to strict data collection techniques that were monitored at specific intervals. Third, statistical packages, such as SPSS (IBM, Armonk, New York), were used for data analysis and to minimize statistical errors.

However, our study has several limitations. First, ultrasound is operator dependent and operator sensitive. Second, transvaginal views are dependent on the volume of urine in the bladder. Third, there is still a stigma related to TVS among pregnant women.

### Conclusion

The inference drawn from our study was that 100% of patients had a shortening of the cervix when measured between 11 to 14 and 18 to 22 weeks of gestation. The evaluation of CL between 18 and 22 weeks of gestation was an inexpensive and sensitive tool for diagnosing PTBs. However, the CL measures during 11 to 14 weeks of gestation were not very sensitive. The reduction in CL was highly sensitive in the prediction of preterm labor. CL measurement can be used with new tools, such as fibronectin measurement, to increase the sensitivity and specificity, making it a better predictor. TVS was used to determine CL measurement at 18 to 22 weeks of gestation and the reduction of CL. Here, TVS was a sensitive and inexpensive test in the prediction of preterm labor.

## CRediT authorship contribution statement

**Nina Mahale:** Project administration, Conceptualization. **Devangi Panchal:** Investigation, Funding acquisition, Formal analysis, Data curation. **Ajit Mahale:** Writing – review & editing, Writing – original draft. **Sonali Ullal:** Resources, Methodology. **Merwyn Fernandes:** Supervision, Software. **Sonali Prabhu:** Visualization, Validation, Supervision.
